# Evaluating Osteogenic Differentiation of Osteoblastic Precursors Upon Intermittent Administration of PTH/IGFBP7

**DOI:** 10.3389/fphar.2022.839035

**Published:** 2022-04-06

**Authors:** Han Xia, Yueyang Tian, Yile Lin, Qia Huang, Yuan Xue

**Affiliations:** Tianjin Key Laboratory of Spine and Spinal Cord, Department of Orthopedic Surgery, Tianjin Medical University General Hospital, Tianjin, China

**Keywords:** parathyroid hormone, anabolic effect, IGFBP7, osteogenic differentiation, bone-forming

## Abstract

Parathyroid hormone (PTH) 1–34 is the first anabolic agent approved for the treatment of osteoporosis. Preclinical evidence shows a potential association between PTH and osteosarcoma. The mechanisms mediating the bone- and neoplasm-forming effects of PTH remain incompleted understood, few studies on the role of Insulin-like growth factor-binding protein 7 (IGFBP7) in mediating the anabolic effects of PTH has been reported. Intermittent PTH administration was found to increase the expression of IGFBP7 in mesenchymal stem cells (MSCs) and pre-osteoblasts. The results indicated that the anabolic effects of PTH were interrupted when knockdown of IGFBP7, while supplementation with IGFBP7 protein could enhance the bone-forming efficacy of PTH and regulate the signaling pathways. Moreover, bone healing was accelerated by the administration of IGFBP7 along with PTH in a mouse model of fracture. The obtained results proved that IGFBP7 was necessary for the anabolic effects of PTH, and combined administration of PTH and IGFBP7 showed stronger bone-forming effects relative to administration of PTH alone.

## Introduction

Parathyroid hormone (PTH) is an 84-amino acid peptide hormone that regulates calcium metabolism and postnatal bone homeostasis (Wein and Kronenberg, 2018). An imbalance in bone turnover associated with weak bone formation and/or excessive resorption results in osteoporosis (Peng et al., 2020). As the first anabolic agent approved for the treatment of osteoporosis (Reeve et al., 1976), teriparatide (PTH1-34) exerts a well-known anabolic effect on bone when administered once daily (Li et al., 2015). Intermittent PTH administration (iPTH) reduces the risk of vertebral and nonvertebral fractures (Neer et al., 2001). The anabolic effect mediated by PTH can be beneficial in implantation fusion and fracture healing (Swarthout et al., 2002). Moreover, PTH may reduce the risk of osteonecrosis (e.g., Kummell’s disease) (Keskinruzgar et al., 2016; Gou et al., 2021).

PTH can directly act on the PTH/PTH-related peptide (PTHrP) receptor (PTHR1) for osteoblastic differentiation (Ishizuya et al., 1997). *In vitro* studies confirm that multiple pathways, including TGF-β/BMP, Wnt/β-catenin, and fibroblast growth factor, transduce in concert to regulate osteogenesis upon iPTH administration (Csukasi et al., 2018; Chen et al., 2021). PTH also increases the osteogenic differentiation of mesenchymal stem cells (MSCs) by enhancing the BMP signaling (Csukasi et al., 2018). Chen et al. also showed that prior activation of Wnt/β-catenin in osteoblasts improves the anabolic effect of PTH (Chen S. et al., 2020). While preclinical evidence showed a potential association between PTH and osteosarcoma (Vahle et al., 2002), the most recent clinical data do not show any such relationship between iPTH and malignancy (Gilsenan et al., 2021). It is crucial to explore the underline mechanisms, which can highlight the process of bone turnover and contribute to the development of osteoporosis treatment and regenerative medicine.

Insulin-like growth factor-binding protein 7 (IGFBP7) is a secretory protein with a low affinity for insulin-like growth factor 1 (IGF-1). IGFBP7 has been implicated as a tumor suppressor (Akiel et al., 2017) and it may protect against bone disease in patients with multiple myeloma (Bolomsky et al., 2015) and promote osteogenesis of MSCs through the Wnt/β-catenin pathway (Zhang et al., 2018). IGFBP7 can induce the osteoblastic switch in the fibroblasts independent of IGF-1 (Degeorges et al., 2000; Lu et al., 2020), which is necessary for the anabolic action of iPTH on the bone. While the activation of the mechanistic target of rapamycin (mTOR) can attenuate the anabolic processes of iPTH, IGFBP7 can protect the proliferative muscle satellite cells by suppressing the activity of the mTOR (Zhang et al., 2017; Chen Z. et al., 2020). The function of IGFBP7 in mediating the bone anabolic effects upon iPTH stimulation remains unclear (Pereira and Canalis, 1999).

In this study, we examined the role of IGFBP7 in mediating the anabolic effect of PTH and evaluated the bone-forming efficacy of combined administration of IGFBP7 and PTH, both *in vitro* and *in vivo*.

## Materials and Methods

### Cell Culture

Mouse pre-osteoblast cell line, MC3T3-E1 clone 12, was cultured in complete media of αMEM (Invitrogen, Camarillo, CA, United States ) supplemented with 10% fetal bovine serum (FBS, Gibco, Camarillo, CA, United States ) and 1% penicillin and streptomycin (Gibco). Mouse bone marrow MSCs at P6-10 (MUBMX-01001; Cyagen Biosciences Inc., Guangzhou, China) were cultured in DMEM (Invitrogen) supplemented with 10% FBS and 1% penicillin and streptomycin (Gibco). The medium was replaced every alternate day. All cells were maintained at 37°C, 5% CO2.

### Experimental Protocol for PTH and IGFBP7 Treatment

The cells were treated with 10 nmol/ml PTH1-34 for the first 0, 3, 4, 5, and 6 h of each 24 h incubation cycle (referred to as 0/24h, 3/24h, 4/24h, 5/24h, and 6/24 h, respectively) for selecting the appropriate mode of iPTH *in vitro*, washed twice with phosphate buffer saline (PBS), and then cultured in the absence of PTH for the remaining duration of the cycle. For exploring the osteogenic effects of IGFBP7, cells were cultured in complete media containing 250 ng/ml IGFBP7 (Sigma-Aldrich, Saint Louis, United States ) (Degeorges et al., 2000; Lu et al., 2020). The impact of IGFBP7 protein on the anabolic effects of iPTH was analyzed by dividing cells into four following groups: control group, IGFBP7 group, PTH group, and PTH + IGFBP7 group. Cells in the IGFBP7 group were cultured in complete media containing 250 ng/ml IGFBP7 on day six to seven, weekly once. Cells were treated with PTH for 4/24 h in the PTH group, while in the PTH + IGFBP7 group, the treatment condition was daily administration of PTH for 4/24 h and supplementation with 250 ng/ml IGFBP7 on day six to seven, weekly once.

### Transfection Using Small Interfering RNAs

Cells were cultured until they reached 50% confluency, following which they were transfected with small interfering RNA (siRNA) for IGFBP7 or the corresponding negative control (NC) siRNA (GenePharma, Suzhou, China) using the Lipofectamine RNAiMAX Reagent (Invitrogen) according to the manufacturer’s instructions. After 4 h of transfection, the media was replaced with fresh complete media.

### Cell Viability Assay

Cells were seeded and cultured in 96-well plates. After supplementation with IGFBP7 protein or transfection with siRNA for 48 h, the cells were treated with 100 μL complete media containing 10 μL cell counting Kit-8 (CCK8, Beyotime, Shanghai, China) reagent for 4 h at 37°C. The absorbance was measured at 450 nm (800 TS; BioTek, United States ).

### Real-Time Quantitative PCR

Total cellular RNA was isolated using the Trizol reagent (Life Technologies, Grand Island, NY, United States ) and reverse transcribed into cDNA using the first-strand cDNA synthesis kit (Thermo Fisher Scientific, Waltham, MA, United States ). All gene transcripts were quantified using TB Green® Premix Ex Taq™ (Takara) on the CFX96 Real-Time Detection System (CFX96; Bio-Rad, CA, United States ) with the following thermal cycling conditions: 95°C for 30 s, followed by 40 cycles of 95°C for 5 s, and 60°C for 30s. The primer sequences are listed in Table 1.

**TABLE 1:** Primer sequences of target genes used in the real time-PCR analysis.

Murine Gene	Primer sequences5′ (-3′)
GAPDH	Forward primer: AGG​TCG​GTG​TGA​ACG​GAT​TTG
	Reverse primer: TGT​AGA​CCA​TGT​AGT​TGA​GGT​CA
IGFBP7	Forward primer: CTG​GTG​CCA​AGG​TGT​TCT​TGA
	Reverse primer: CTC​CAG​AGT​GAT​CCC​TTT​TTA​CC
Runx2	Forward primer: GAC​TGT​GGT​TAC​CGT​CAT​GGC
	Reverse primer: ACT​TGG​TTT​TTC​ATA​ACA​GCG​GA
Sp7	Forward primer: ACC​CCA​AGA​TGT​CTA​TAA​GCC​C
	Reverse primer: CGC​TCT​AGC​TCC​TGA​CAG​TTG
ALP	Forward primer: CCA​ACT​CTT​TTG​TGC​CAG​AGA
	Reverse primer: GGC​TAC​ATT​GGT​GTT​GAG​CTT​TT
Bglap	Forward primer: GAA​CAG​ACA​AGT​CCC​ACA​CAG​C
	Reverse primer: TCA​GCA​GAG​TGA​GCA​GAA​AGA​T
COLA1	Forward primer: CGA​TGG​ATT​CCC​GTT​CGA​GT
	Reverse primer: GCT​GTA​GGT​GAA​GCG​ACT​GT
Akt1	Forward primer: ATG​AAC​GAC​GTA​GCC​ATT​GTG
	Reverse primer: TTG​TAG​CCA​ATA​AAG​GTG​CCA​T
Mtor	Forward primer: CAG​TTC​GCC​AGT​GGA​CTG​AAG
	Reverse primer: GCT​GGT​CAT​AGA​AGC​GAG​TAG​AC
BMP2	Forward primer: GGG​ACC​CGC​TGT​CTT​CTA​GT
	Reverse primer: TCA​ACT​CAA​ATT​CGC​TGA​GGA​C
β-catenin	Forward primer: ATG​GAG​CCG​GAC​AGA​AAA​GC
	Reverse primer: CTT​GCC​ACT​CAG​GGA​AGG​A

### Western Blotting

Proteins were extracted using RIPA buffer (Beyotime) supplemented with 1% phosphatase inhibitor and 1% phenylmethanesulfonyl fluoride. Proteins were loaded and separated on 10% SDS-PAGE gels and transferred onto polyvinylidene fluoride membranes for immunoblotting. The following antibodies were used: GAPDH (1/10000; Abcam, Cambridge, United States ), Runt-related transcription factor 2 (Runx2, 1/1000; Abcam), osterix (Sp7, 1/1000; Abcam), type I collagen (COL1, 1/1000; Abcam), and *β*-catenin (1/1000; CST, United States ). The membranes were incubated with horseradish peroxidase-conjugated goat anti-rabbit IgG (1/10000; GenScript, United States ) for 1 h. The blot was scanned on the GelView 6000PLus Smart Gel Imaging System (BLT, Guangzhou, China). The quantification of protein levels was calculated as a ratio of the protein of interest to that of the loading control (GAPDH).

### Staining Alkaline Phosphatase

Cells were fixed using 4% paraformaldehyde at 4°C for 30 min, rinsed thrice with deionized water, and incubated in alkaline phosphatase (ALP) staining solution (Beyotime) for 1 h in the dark. Images were captured using a microscope after rinsing the samples in deionized water for 5 min. ALP can catalyze the formation of insoluble chromogenic substrates; the ALP activity was assessed at 405 nm.

### Staining Alizarin Red S

Calcium deposition was assessed by Alizarin red staining (ARS) on day 14. Cells were fixed by immersion in 70% ice-cold ethanol at 4°C for 1 h and washed thrice with PBS. After incubation in 40 mM Alizarin red S solution (pH 4.2) for 15 min and washing thrice with deionized water, the images were captured under a microscope. The area of red staining was quantified using ImageJ.

### *In Vivo* Experiments

The animal experimental protocol detailed below was performed in accordance with the Laboratory Animal Ethics Committee of the Institute of Radiation Medicine, Chinese Academy of Medical Sciences. The fracture model was established as described in previous studies (Shi et al., 2018; Guo et al., 2019). The bone defect gap was 0.8 mm and the tibia on the fracture side was fixed by inserting an intramedullary needle of 0.35 mm diameter. Two-month-old male C57-BL mice, with an average weight of 21 ± 3 g, were randomly divided into four groups (*n* = 5 per group) as follows: 1) mice in the control group were treated with PBS; 2) mice in the PTH group were administered PTH 40ug/kg/d subcutaneously; 3) mice in the IGFBP7 group were intravenously administered IGFBP7 (2 μg IGFBP7 in 100 μL PBS, once a week, based on previous studies) into the tail vein (Bolomsky et al., 2015; Ye et al., 2020), and 4) mice in the PTH + IGFBP7 group were administered PTH 40ug/kg/d subcutaneously and IGFBP7 (2 μg IGFBP7 in 100 μL PBS, once a week) through the tail vein.

### Microcomputed Tomography Scanning

Mice were euthanized and sacrificed 20 days after surgery. Samples were collected after removal of the intramedullary needle and fixed in 4% paraformaldehyde for micro-computed tomography (micro-CT) scanning (SkyScan 1276; SkyScan, Kartuizersweg, Belgium). All samples were scanned using the following settings: voxel size, 12 μm; 80 kVp; 200 μA; 0.25-mm AL filter, and integration time, 400 m. Mice were excluded from the study in cases of failure of intramedullary needle removal (one mouse in the control group).

### Histological Evaluation

The samples were fixed in 4% paraformaldehyde and decalcified with 10% ethylenediaminetetraacetic acid (EDTA, Sigma). Serial sections were cut with slices of 3 μm thickness. H&E staining, safranin O-fast green staining for cartilage and bone matrix (FG), and immunohistochemical staining were performed separately on consecutive tissue sections. The following antibodies were used for immunohistochemical staining: COL1 (1:200; Bioss, Beijing, China) and OCN (1:200; Bioss, Beijing, China) and goat anti-rabbit IgG secondary antibody (HRP, 1:100; Sino biological, Beijing, China). Images were captured under a microscope**.**

### Statistical Analysis

Each experiment was carried out for at least triplicates. Statistical analysis was performed using the SPSS software (version 21.0; IBM, Armonk, United States ). Data were presented as mean ± SD. The two-tailed Student’s t-test or one-way ANOVA followed by a post hoc test was used for comparisons. Statistical significance was set at *p* < 0.05.

## Results

### PTH Improves the Expression of Osteo-Related Marker Genes and IGFBP7 in BMSCs and MC3T3-E1

**FIGURE 1:**
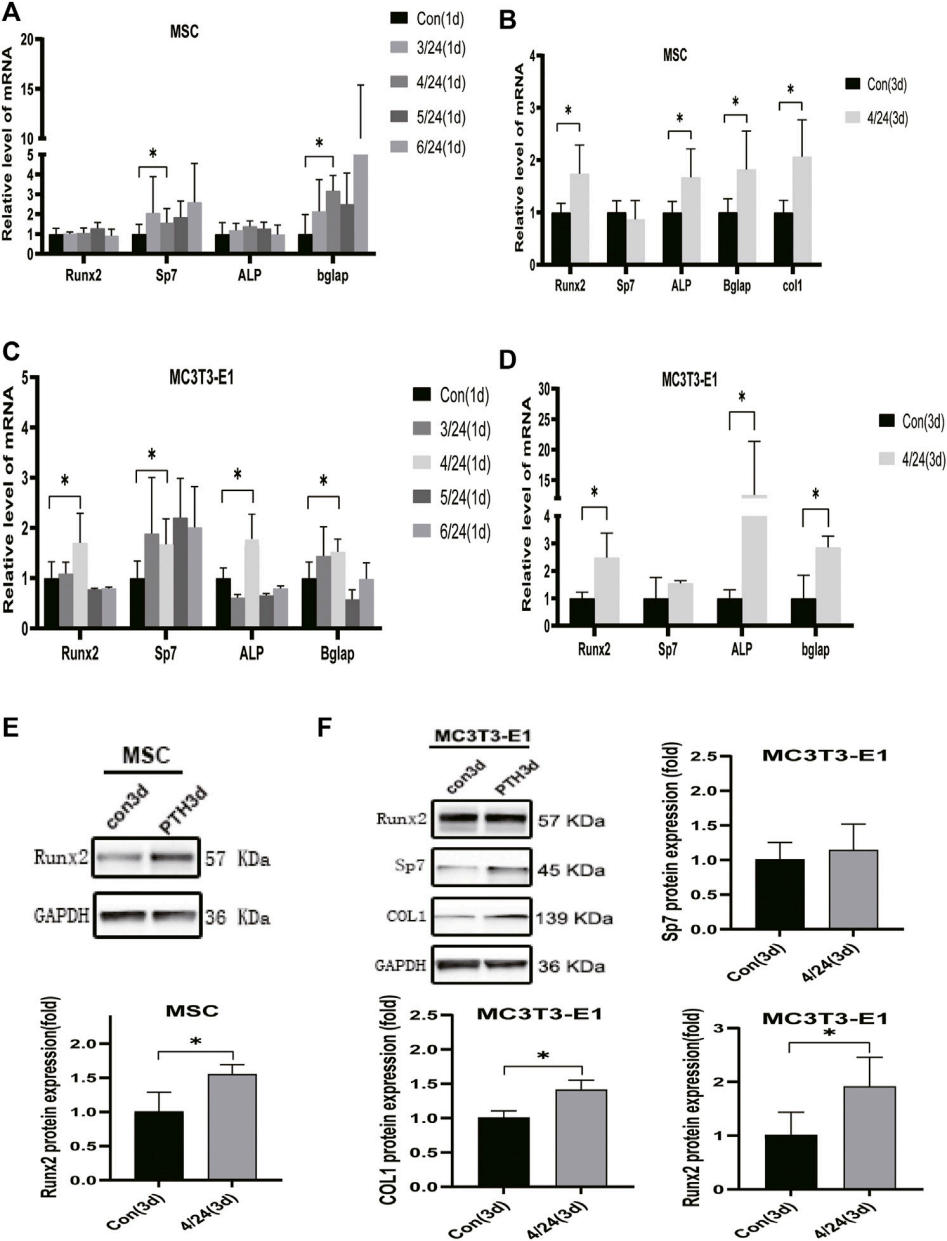
Effects of iPTH on osteogenic differentiation of MSCs and MC3T3-E1 cells. **(A)** Relative mRNA expression levels of osteogenic marker genes on d one in MSCs upon treatment with 10 nmol/ml PTH1-34 for 0/24h, 3/24h, 4/24h, 5/24h, and 6/24 h. **(B)** Relative mRNA expressions of osteogenic marker genes on d three in MSCs upon iPTH for 4/24 h. **(C)** Relative mRNA expressions of osteogenic marker genes on d one in MC3T3-E1 cells upon iPTH for 0/24h, 3/24h, 4/24h, 5/24h, and 6/24 h. **(D)** Relative mRNA expressions of osteogenic marker genes on d three in MC3T3-E1 cells upon iPTH for 4/24 h. **(E)** Relative protein expression of Runx2 on d three in MSCs upon iPTH for 4/24 h. **(F)** Relative protein expressions of osteogenic markers on d three in MC3T3-E1 cells upon iPTH for 4/24 h. *p < 0.05.

**FIGURE 2:**
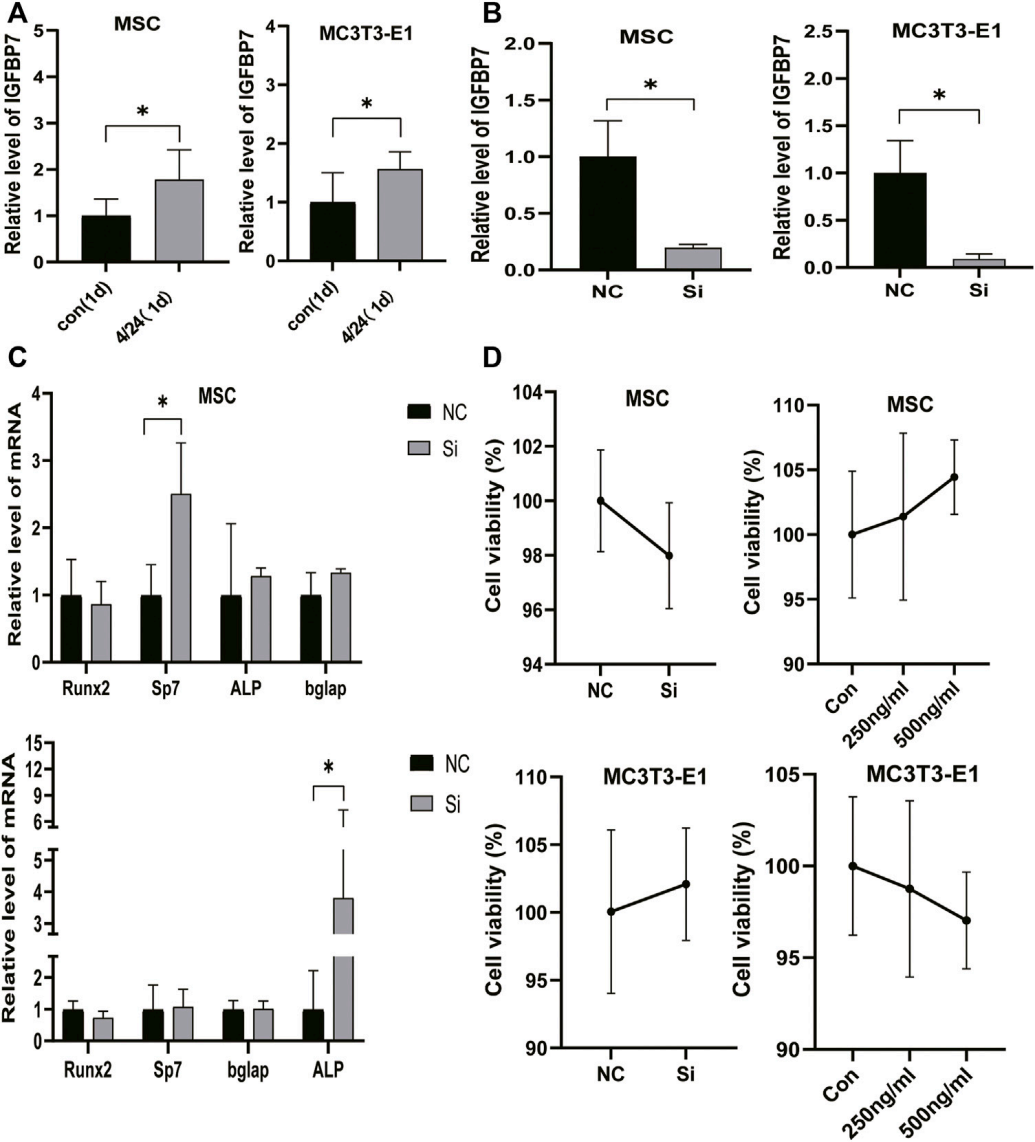
Verification of IGFBP7 knockdown in BMSCs and MC3T3-E1 cells. **(A)** The expression of IGFBP7 mRNA induced by iPTH for 4/24 h on d one in BMSCs and MC3T3-E1 cells. **(B)** Establishment of IGFBP7 knockdown in BMSCs and MC3T3-E1 cells. **(C)** Relative mRNA expressions of osteogenic marker genes at 48 h after transfection in BMSCs and MC3T3-E1 cells in the negative control group (NC) and IGFBP7 silenced group (Si). **(D)** BMSCs and MC3T3-E1 cell proliferation was examined by the CCK-8 assay. *p < 0.05.

PTH exerts an anabolic effect in a dose- and time-dependent manner. Analysis of qRT-PCR showed that in MSCs, iPTH treatment for 4/24 h could significantly increase the mRNA expression of Sp7, Bglap on day 1 (Figure 1A) and that of Runx2, Sp7, ALP, Bglap, and COL1 on day 3 (Figure 1B); in MC3T3-E1 cells, the mRNA expression of Runx2, Sp7, ALP, and Bglap on day 1 (Figure 1C) and that of Runx2, ALP, Bglap on day 3 were significantly upregulated after iPTH treatment for 4/24 h (Figure 1D). Analysis of western blotting showed that iPTH treatment for 4/24 h could significantly increase the protein expression of Runx2 in MSCs (Figure 1E) and that of Runx2 and COL1 in MC3T3-E1 cells (Figure 1F) on day 3. The gene expression of IGFBP7 (Figure 2A) increased after iPTH treatment for 24 h in both MSCs and MC3T3-E1 cells relative to the control group.

### IGFBP7 Knockdown in BMSCs and MC3T3-E1 Cells

SiRNA constructs targeting IGFBP7 (5-GAG​UAU​GAG​UGC​CAC​GCA​UTT-3) (si group) and NC-siRNA (NC group) were used for transfection. The levels of IGFBP7 mRNA decreased significantly in comparison with the control group in MSCs and MC3T3-E1 cells (Figure 2B).

Supplementation of IGFBP7 Protein or Knocking Down IGFBP7 does not Influence the Proliferation of MSCs and MC3T3-E1 Cells.

IGFBP7 can reduce the survival of AML cells by inducing cell cycle arrest in the G2 phase and apoptosis (Verhagen et al., 2014). The CCK-8 assay was performed to assess whether the supplementation of IGFBP7 protein or knocking down IGFBP7 for 2 days influenced the cell viability. There were no significant differences in cell viability after supplementation with IGFBP7 protein or knocking down IGFBP7 for 2 days. (Figures 2E, F).

### IGFBP7 Protein Enhances the Anabolic Effect of iPTH

**FIGURE 3:**
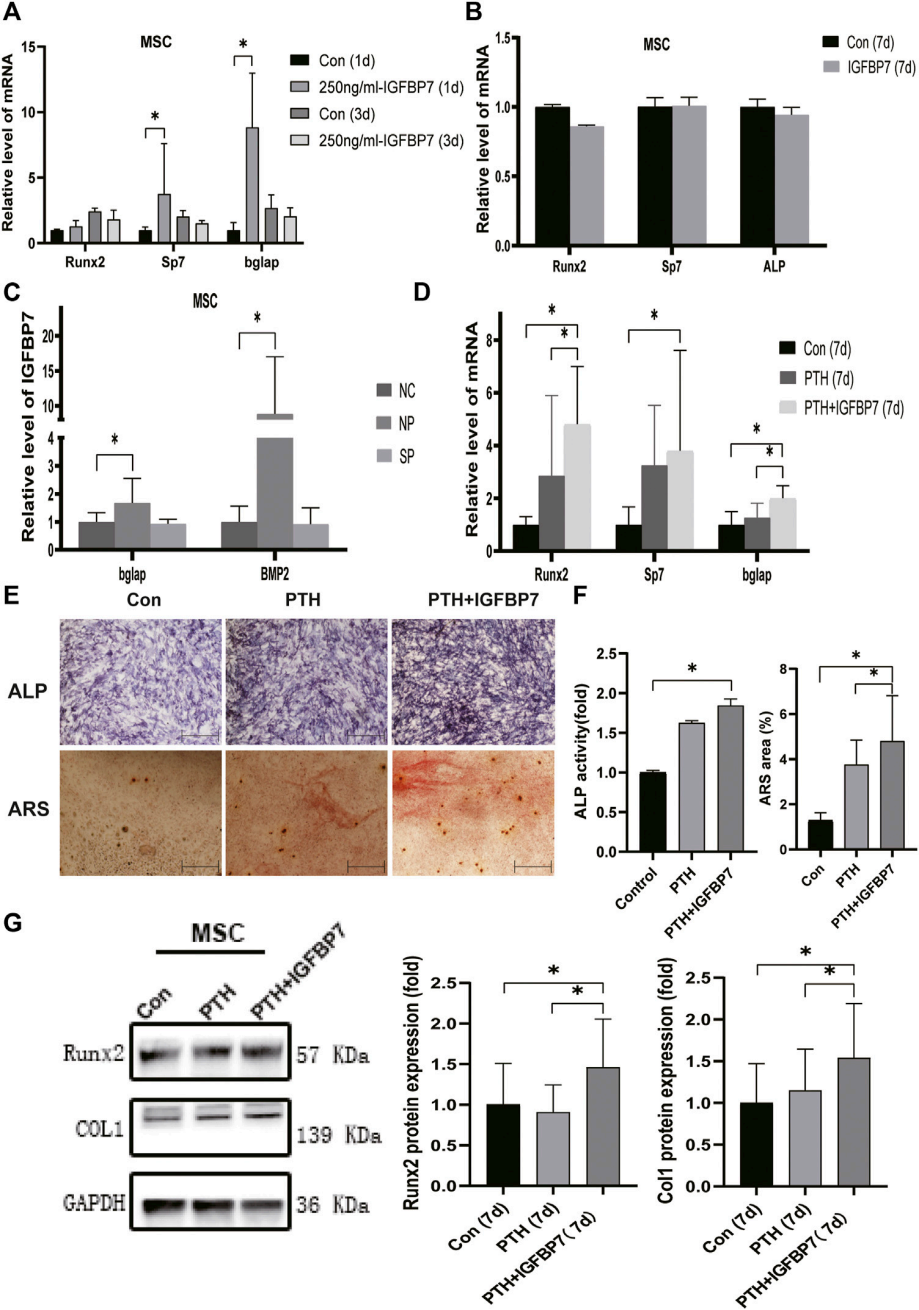
Impacts of IGFBP7 on osteogenic differentiation of MSCs induced by iPTH. **(A)** The mRNA expression of osteogenic marker genes induced by250 mg/ml IGFBP7 for d one and d 3. **(B)** Relative mRNA expressions of osteogenic marker genes in IGFBP7 group on d7. **(C)** Relative mRNA expressions of osteogenic marker genes induced by iPTH for 4/24 h on d 1 after transfection in BMSCs in the negative control group (NP) and IGFBP7 silenced group (SP). **(D)** Relative mRNA expressions of osteogenic marker genes induced by iPTH alone (PTH group) and IGFBP7 combined with PTH (PTH + IGFBP7 group) for 7 days. **(E)** ALP staining on day 7; Scale bars, 400 μm. Alizarin red staining on day 14; Scale bars, 400 μm. **(F)** quantification of ALP activity and calcium deposition induced by iPTH alone (PTH group) and IGFBP7 combined with PTH (PTH + IGFBP7 group). **(G)** Relative protein expressions of Runx2 and COL1 in MSCs induced by iPTH alone (PTH group) and IGFBP7 combined with PTH (PTH + IGFBP7 group) for 7 days *p < 0.05.

**FIGURE 4:**
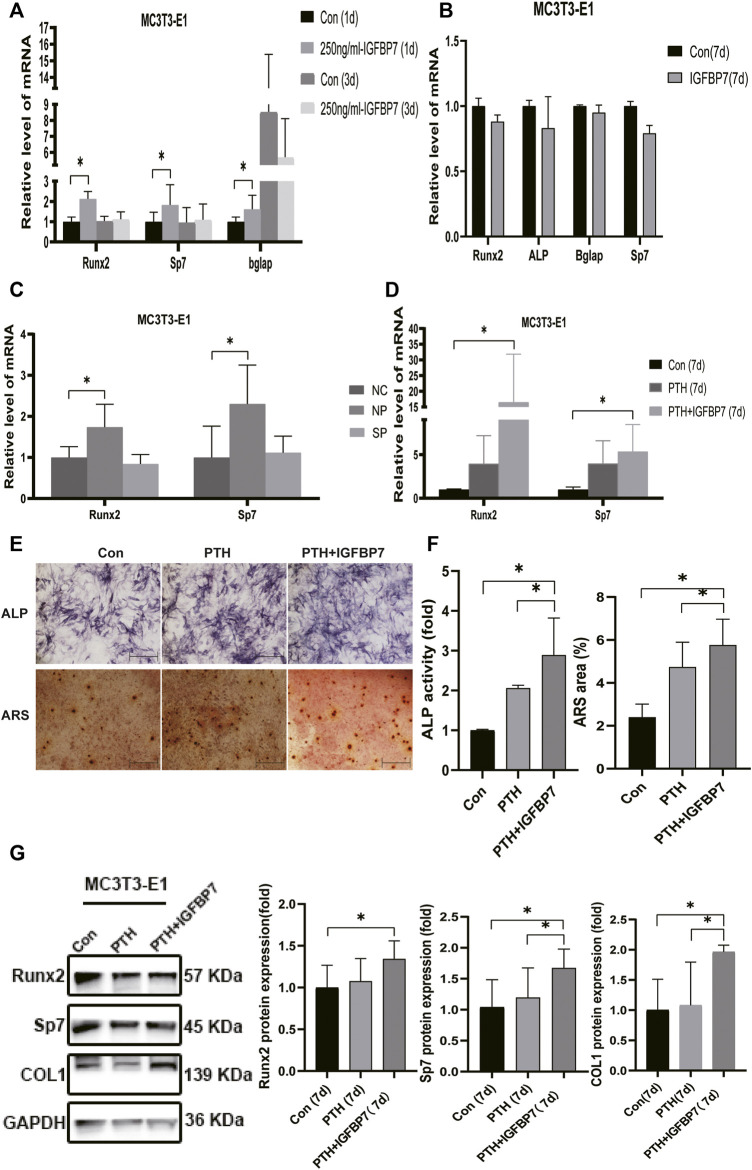
Impacts of IGFBP7 on osteogenic differentiation of MC3T3-E1 cells induced by iPTH. **(A)** The mRNA expression of osteogenic marker genes induced by 250 mg/ml IGFBP7 for d one and d 3. **(B)** Relative mRNA expressions of osteogenic marker genes in IGFBP7 group on d7. **(C)** Relative mRNA expressions of osteogenic marker genes induced by iPTH for 4/24 h on d 1 after transfection in MC3T3-E1 cells in the negative control group (NP) and IGFBP7 silenced group (SP). **(D)** Relative mRNA expressions of osteogenic marker genes induced by iPTH alone (PTH group) and IGFBP7 combined with PTH (PTH + IGFBP7 group) for 7 days. **(E)** ALP staining on day 7; Scale bars, 400 μm. Alizarin red staining on day 14; Scale bars, 400 μm. **(F)** quantification of ALP activity and calcium deposition induced by iPTH alone (PTH group) and IGFBP7 combined with PTH (PTH + IGFBP7 group). **(G)** Relative protein expressions of Runx2, Sp7, and COL1 in MC3T3-E1 cells induced by iPTH alone (PTH group) and IGFBP7 combined with PTH (PTH + IGFBP7 group) for 7 days *p < 0.05.

Supplementation with IGFBP7 significantly increased the mRNA expression of Sp7, and Bglap in MSCs and that of Runx2, Sp7, and Bglap in the MC3T3-E1 cells on day 1 relative to the control group. There were no significant differences in the gene expression levels of Runx2, Sp7, and Bglap in both MSCs (Figure 3A) and MC3T3-E1 cells (Figure 4A) after supplementation with IGFBP7 protein for 3 days as compared to those in the control group. No increased expression of osteo-specific genes in both MSCs (Figure 3B) and MC3T3-E1 cells (Figure 4B) were detected in the IGFBP7 group on d7 as compared to the control group. These results indicated that long-term addition of IGFBP7 without iPTH failed to induce osteogenic differentiation.

In MSCs: the mRNA expression levels of Runx2, Sp7, and Bglap were significantly higher in the PTH + IGFBP7 group on day 7 relative to the control and PTH groups (Figure 3D); increased ALP activity was observed in the PTH and PTH + IGFBP7 groups on day 7 as compared to the control group; more calcium deposition was detected in the PTH + IGFBP7 group as compared to the control and PTH groups on day 14 (Figures 3E, F); the protein expression levels of Runx2 and COL1 were significantly higher in the PTH + IGFBP7 group relative to the control and PTH groups on day 7 (Figure 3G).

In MC3T3-E1 cells: the mRNA expression levels of Runx2 and Sp7 were significantly higher on day 7 in the PTH + IGFBP7 group in comparison with the control and PTH groups (Figure 4D); increased ALP activity was observed in the PTH + IGFBP7 group on day 7 as compared to that in the control and PTH groups; more calcium deposition was detected in the PTH + IGFBP7 group as compared to the control and PTH groups on day 14 (Figures 4E, F); the protein expression levels of COL1 and Sp7 were significantly higher in the PTH + IGFBP7 group as compared to the control and PTH groups on day 7 (Figure 4G).

Knockdown of IGFBP7 Reduces the Expression of Osteo-related Genes upon iPTH.

The influence of silencing IGFBP7 on the anabolic effects of iPTH was assessed. The cells were divided into the following four groups: NC group, Si group, NP group, and SP group. Cells were transfected with the NC siRNA in the NC group or the siRNA for IGFBP7 in the Si group. Cells were treated with PTH for 4/24 h after transfection with the NC siRNA in the NP group or the siRNA for IGFBP7 in the SP group for 4 h and replaced with fresh complete media. Knockdown of IGFBP7 increased the expression of Sp7 mRNA in MSCs and ALP mRNA in MC3T3-E1 cells on day 1, which may be related to the potential effects of IGFBP7 on the cell cycle (Verhagen et al., 2014). There were no significant differences in the mRNA expressions of Runx2, ALP, and Bglap in MSCs and Runx2, Sp7, and Bglap in MC3T3-E1 cells after knockdown of IGFBP7 for 1 day (Figure 2C). A lower mRNA expression level of Bglap was induced by iPTH at 24 h in MSCs of the SP group as compared to the NP group (Figure 3C). In MC3T3-E1 cells, lower mRNA expression levels of Runx2 and Sp7 were induced upon iPTH at 24 h in the SP group as compared to the NP group (Figure 4C).

### IGFBP7 Regulates mTOR, TGF-β/BMP, and Wnt/β-Catenin Signaling Pathways

**FIGURE 5:**
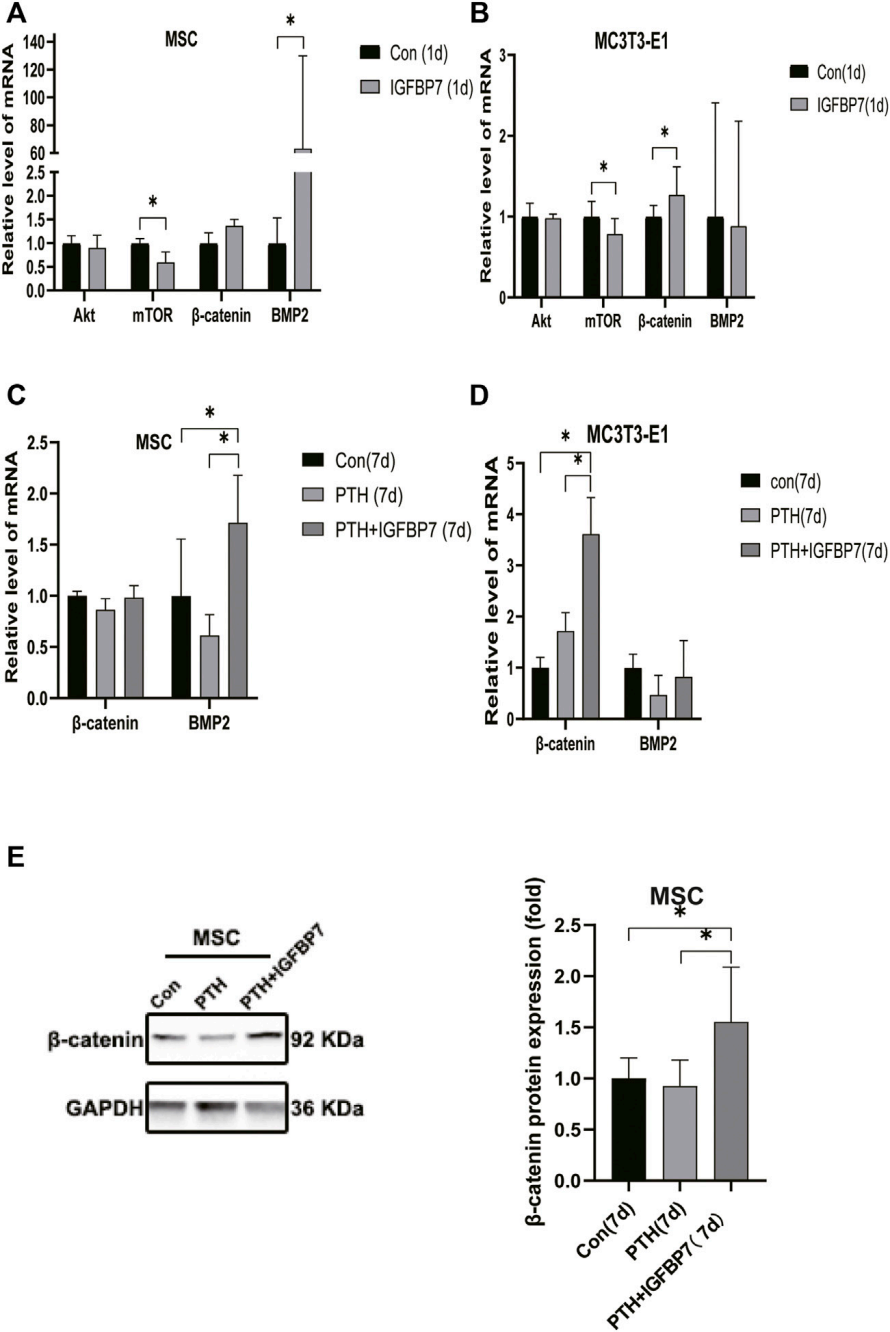
Supplementation of IGFBP7 protein regulates the expression of mTOR, BMP2 and *β*-catenin. **(A)** The mRNA expression levels of Akt, mTOR, BMP2 and *β*-catenin in MSCs induced by supplementation using IGFBP7 for 24 h. **(B)** Relative mRNA expressions of Akt, mTOR, BMP2 and *β*-catenin in MC3T3-E1 cells induced by supplementation of IGFBP7 for 24 h. **(C)** Relative mRNA expression of *β*-catenin and BMP2 in MSCs induced by iPTH alone (PTH group) and IGFBP7 combined with PTH (PTH + IGFBP7 group) for 7 days. **(D)** Relative mRNA expression of *β*-catenin and BMP2 in MC3T3-E1 cells induced by iPTH alone (PTH group) and IGFBP7 combined with PTH (PTH + IGFBP7 group) for 7 days. **(E)** Relative expression of the *β*-catenin protein in MSCs induced by iPTH alone (PTH group) and IGFBP7 combined with PTH (PTH + IGFBP7 group) for 7 days *p < 0.05.

Supplementation with IGFBP7 protein was found to reduce the expression of the mechanistic target of rapamycin kinase (mTOR) mRNA and increase the expression of *β*-catenin gene on d1 in both MSCs and MC3T3-E1 cells (Figures 5A, B). The addition of IGFBP7 also increased the expression of BMP2 mRNA in MSCs on day 1 (Figure 5A), and the level of BMP2 gene induced by iPTH decreased on d1 when silencing of IGFBP7 in MSCs (Figure 3B). IGFBP7 protein combined with PTH significantly increase the expression of *β*-catenin mRNA on day 7 relative to iPTH alone in MC3T3-E1 cells (Figure 5D). Unlike MSCs, no increase in the mRNA expression of BMP2 in MC3T3-E1 cells was detected after supplementation with IGFBP7 for 1 day or combined administration of PTH and IGFBP7 for a week relative to the control group. There were no differences between the expressions of *β*-catenin mRNA in PTH and PTH + IGFBP7 groups in MSCs on day 7. IGFBP7 protein combined with PTH significantly upregulated the expression of BMP2 mRNA (Figure 5C) and protein expression level of *β*-catenin on day 7 relative to iPTH alone in MSCs (Figure 5E). These data indicated that IGFBP7 may inhibit the mTOR pathway and enhance TGF-β/BMP and Wnt-β-catenin signaling cascades.

### IGFBP7 Protein Combined With PTH Accelerate Bone Healing in the Mouse Model of Fracture

**FIGURE 6:**
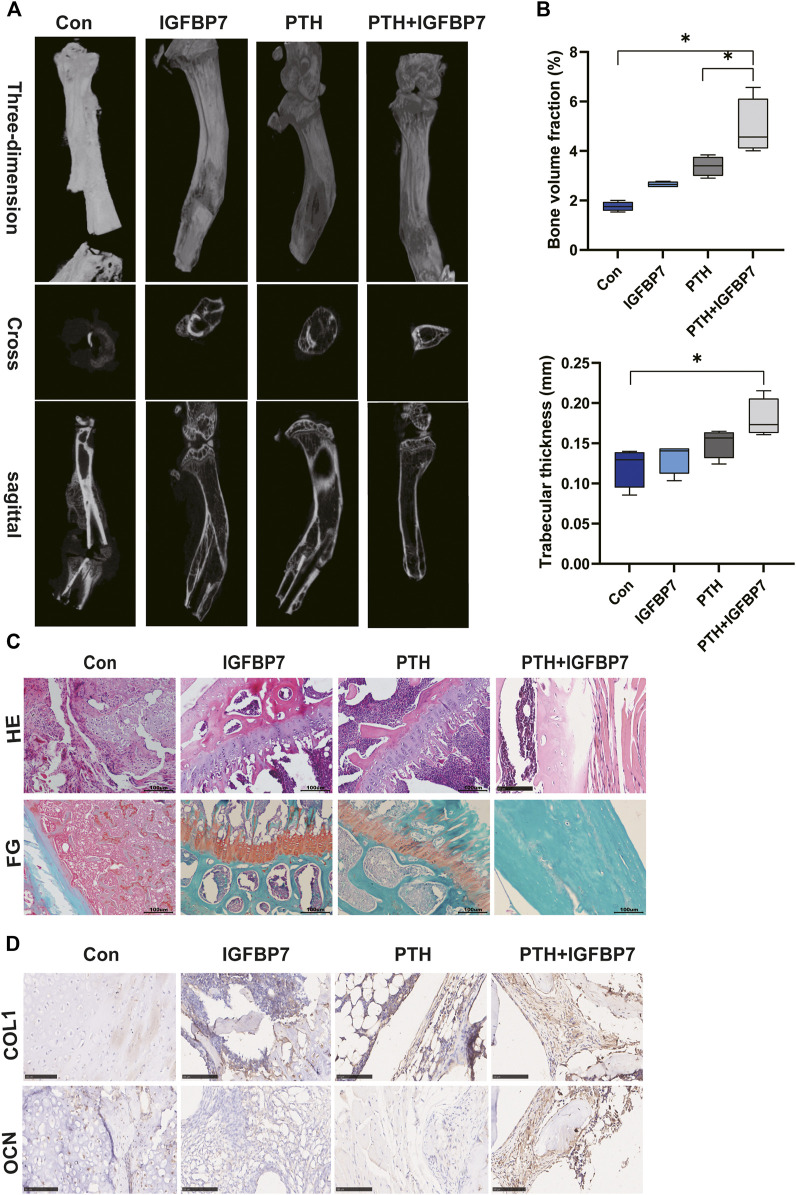
IGFBP7 combined with PTH enhances bone healing in a mouse fracture model. **(A)** Images of the bone defect sites detected by micro-CT scanning. **(B)** Bone volume fraction and trabecular thickness were analyzed. *p < 0.05. **(C)** Histological analysis for bone healing, H&E; fast green and Safranin staining, FG; Scale bars, 100 μm. **(D)** Immunohistochemical staining for COL1 and OCN in bone healing sites; Scale bars, 100 μm.

Micro-CT scanning showed that bone formation increased significantly in the PTH + IGFBP7 group as compared to the control and PTH groups. The bone defect gap was the smallest in the PTH + IGFBP7 group as compared to the other groups (Figure 6A). Quantitatively, the bone volume fraction was significantly higher in the PTH + IGFBP7 group as compared to the other groups. A significant increase in the trabecular thickness was found in the PTH and PTH + IGFBP7 groups as compared to the control group (Figure 6B). Histological staining showed that administration of IGFBP7 combined with PTH significantly enhanced collagen deposition as compared to iPTH alone (Figure 6C). Higher levels of OCN and COL1 proteins were found in the PTH + IGFBP7 group relative to the other groups (Figure 6D).

## Discussion

Osteoporosis results from an imbalance in bone turnover associated with weak bone formation and excessive resorption (Eastell and Szulc, 2017). Most treatment approaches for osteoporosis reduce bone resorption rather than enhance the bone formation (Gregson et al., 2020; Peng et al., 2020). As the first anabolic agent approved for the treatment of osteoporosis, PTH stimulates the proliferation and osteogenic differentiation of osteoblast precursor cells (Swarthout et al., 2002).

*In vitro* studies show that PTH has an osteoanabolic function that is dose-and time-dependent. In this study, MSCs and MC3T3-E1 cells were treated with PTH1-34 for the first few hours of each 24 h incubation cycle, and the expression of osteo-related genes, including Runx2, Sp7, and Bglap, were found to increase after PTH treatment for 4/24 h. Moreover, increased ALP activity and calcium deposition were observed after iPTH for 4/24 h. Runx2 is a critical osteogenic transcription factor that is expressed during the osteogenic differentiation (Baniwal et al., 2012). The augmentation of osteoblast differentiation is closely related to the high expression of the key osteoblastic marker genes, namely ALP, Sp7, and Bglap (Watts, 1999). Results at single-cell resolution suggest that the expression of Bglap is upregulated along with the progression of the osteogenic differentiation (Tikhonova et al., 2019). Accordingly, we confirmed that MSCs and MC3T3-E1 cells osteogenic differentiated after PTH treatment for 4/24 h.

Despite PTH exerting an anabolic effect on bone *in vivo*, the underlying mechanisms are not completely understood. Although PTH1-34 does not increase the incidences of adult osteosarcoma as reported in a 15-years US post-marketing surveillance study (Gilsenan et al., 2021), the blunting of bone-forming efficacy and potential carcinogenic effects after iPTH for 2 years has hampered the development of osteoporosis combination therapies (Black and Rosen, 2016; Eriksen and Brown, 2016; Chen S. et al., 2020). As a tumor suppressor, IGFBP7 can protect patients against bone disease in cases of multiple myeloma and overcome activin A-induced osteoblast suppression along with promoting osteogenesis *in vitro* (Bolomsky et al., 2015). Zhang et al., report that IGFBP7 regulates the osteogenic differentiation of MSCs via the Wnt/β-catenin signaling pathway (Zhang et al., 2018). Lu et al., also report that IGFBP7 can induce osteogenic differentiation of fibroblasts (Zhang et al., 2018; Lu et al., 2020). Moreover, Ye et al., show that IGFBP7 can inhibit receptor activation of nuclear factor-κB ligand (RANKL)-induced osteoclastogenesis (Ye et al., 2020). The regulatory role of IGFBP7 on the osteoanabolic effect of iPTH has been rarely reported. To the best of our knowledge, this is the first study to examine the role of IGFBP7 in the anabolic effects of PTH. We found that iPTH not only promoted osteogenic differentiation but also elevated the expression of IGFBP7 in MSCs and MC3T3-E1 cells. Supplementation with recombinant IGFBP7 protein (250 ng/ml) increased the expression of osteogenic-specific genes in both MSCs and MC3T3-E1 cells. Moreover, supplementation with IGFBP7 significantly increased the expression of osteogenic-specific genes and calcium deposition induced by iPTH, while the knockdown of IGFBP7 decreased the expression of osteogenic-specific genes induced by iPTH in MSCs and MC3T3-E1 cells.

Multiple signaling pathways mediate the anabolic effects of iPTH, including the Wnt/β-catenin and TGF-β/BMP pathways (Chen et al., 2021). Recent studies show that mTOR complex 1 (mTORC1) activation downregulates FGFR3 and PTHR1 in articular chondrocytes and initiates osteoarthritis (Zhang et al., 2017). Decreased activities of mTORC1 and mTOR complex 2 (mTORC2) have been observed in a disorder caused by constitutive activation of the PTH/PTHrP receptor (Csukasi et al., 2018). We found that supplementation with IGFBP7 protein reduced the mRNA expression of mTOR, the catalytic subunit of mTORC1 and mTORC2, and increased the mRNA expression of *β*-catenin in both MSCs and MC3T3-E1 cells. Supplementation with IGFBP7 also increased the expression of BMP2 mRNA in MSCs, while the mRNA expression of BMP2 induced by iPTH decreased upon IGFBP7 knockdown. Moreover, IGFBP7 protein combined with PTH administration improved the expression of *β*-catenin mRNA in MC3T3-E1 cells and *β*-catenin protein in MSCs relative to iPTH alone. These data indicated that IGFBP7 inhibited the mTOR pathway and enhanced Wnt/β-catenin and TGF-β/BMP signaling cascades.

The anabolic effect mediated by PTH can be sustained throughout the process of fracture healing (Swarthout et al., 2002). Bone formation and bone mineralization increase significantly within calvarial defects upon daily administration of PTH (Weng et al., 2019). In our study, IGFBP7 combined with PTH accelerated bone healing in a mouse model of fracture, with increased bone formation and collagen deposition detected by micro-CT and histological analyses.

However, this study has some limitations. First, although the results showed that IGFBP7 effectively enhanced the anabolic effect of iPTH, the safety of IGFBP7 in humans was not evaluated. Second, we did not use transgenic mice. Third, although IGFBP7 can reduce osteoclastogenesis induced by RANKL (Ye et al., 2020), we did not explore the impact of IGFBP7 on the suppression of the bone catabolic effect of PTH. Fourth, although IGFBP7 has been implicated as a tumor suppressor, we did not evaluate the function of IGFBP7 in reducing the carcinogenic effects of PTH, and further studies are needed to address these limitations.

In conclusion, the study revealed that silencing of IGFBP7 interrupted the anabolic effects of PTH, and combined administration of PTH and IGFBP7 showed stronger bone-forming effects than PTH alone. These results indicated that IGFBP7 is necessary for the osteoanabolic effects of PTH and may provide novel insight into bone tissue regeneration.

## Data Availability

The raw data supporting the conclusion of this article will be made available by the authors, without undue reservation.
